# First Record of the Carmine Spider Mite, *Tetranychus urticae*, Infesting *Withania somnifera* in India

**DOI:** 10.1673/031.012.5001

**Published:** 2012-04-10

**Authors:** Ashutosh Sharma, Pratap Kumar Pati

**Affiliations:** Department of Biotechnology, Guru Nanak Dev University, Amritsar-143005, Punjab, India

**Keywords:** Ashwagandha, medicinal plant, pest management, Tetranychidae

## Abstract

During April–June 2010, red two—spotted carmine spider mites *Tetranychus urticae* Koch (Trombidiformes: Tetranychidae) were found on aerial apical parts of Ashwagandha *Withania somnifera* (L.) Dunal (Solanales: Solanaceae) plants in the Amritsar District of Punjab Province in the North Indian plains. The mites fed on the leaves, making them shiny white in color, which gradually dried off and were later shed. The pest was identified as *T. urticae*. To best of our knowledge, this is the first record of this pest infesting *W. somnifera* in India.

## Introduction

*Withania somnifera* (L.) Dunal (Solanales: Solanaceae) is a traditional Indian medicinal plant of high repute, prone to a number of diseases and pests both under wild and cultivated conditions (Nagraj and Reddy 1985; Gupta et al. 1993; Pati et al. 2008; Sharma et al. 2011). Extracts from different plant parts including root, shoot, leaves, seed, and berry have been commonly used in various remedies. Recently, the leaf extracts of *Withania* have been explored for its strong anti—cancer activity and selective killing of cancer cells, which is attributed its constituent Withanone that activates the tumor suppressor protein p53 in cancer cells only (Widodo et al. 2010).

## Materials and Methods

During the present investigation of pests infesting *W. somnifera* in Amritsar region of Punjab province, carmine spider mite *Tetranychus urticae* Koch (Trombidiformes: Tetranychidae) (Smith-Meyer 1974) was identified as one of the most potent pests harming the overall plant growth and vigor both in field grown and wild populations of *W. somnifera*. The plants were grown in Net— house at the Department of Biotechnology, Guru Nanak Dev University, Amritsar (74.82323–74.82332 °E, 31.63678–31.63688 ° N, 221 MASL). The pests were collected from infested leaves and photographed using Sony Cyber—Shot DSC W350 (www.sony.com) and stereo—microscope (Olympus, www.olympus.com) coupled Magnus image capturing system.

## Results

**Figure 1.**
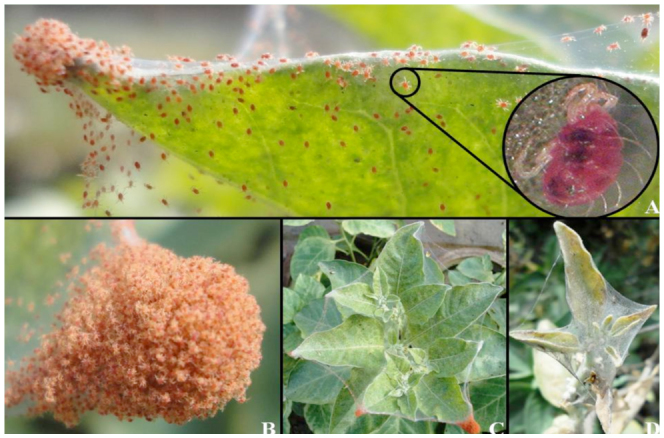
*Withania somnifera* plant infested by red spider mite *Tetranychus urticae*. (A) Heavily infested leaf with a single spider mite is zoomed out in spherical window. (B) Swarm of mites on a leaf tip. (C) Apical young leaves covered by web like structures. (D) Damaged apical part after heavy infestation. High quality figures are available online.

To the best of our knowledge, there has not been a record of *T. urticae* infesting *W. somnifera*. *Tetranychus urticae* feeds on the leaves of *W. somnifera* during the dry and hot periods of the year (April–June) when average maximum temperature rises to 35 °C or more. They infest the greenhouse plants in relatively cooler periods of the year. It generally feeds on the lower side of the leaves and forms spider web—like structures; the huge swarms of the mites on leaf tips are visible to naked eye. They generally feed on the plants growing in strong sunlight and are most virulent during hot and dry periods of the year (April–June). The pest leaves behind white and silvery spots on leaves after feeding on them. They penetrate the leaf tissues with the help of cheliceral—stylets and then suck in the liberated plant fluid; subsequent removal of chlorophyll and plant pigments usually lessens their green appearance, which is referred to as blotching of the leaves (Figure 1D). During severe attack, leaves gradually turn gray— white, sometimes light brown and ensuing sudden complete leaf fall. The mites spread in whole field and may lead to 90–100% defoliation of the leaves. Its nymphs are numerous, fast moving, pink—red colored, and can cover the entire top of the plant, especially the tips of young leaves (Figures 1B, 1C). Adults are relatively dark purple—red in color, with two dark spots on their body (Figure 1A). During heavy infestations, the mites produce a web all over the plant apex (Figure 1C, 1D). The mites develop very rapidly on its host plants causing distress and quick leaf fall, and ultimately leading to death of the plants. The pest is transmitted to adjacent uninfested plants by spider web/net—like structures created by the mites, connecting the leaves of adjacent plants.

## Discussion

*Tetranychus urticae* is found on a wide range of hosts like groundnut, vegetable crops, and fruit trees (Nandagopal and Gedia 1995), but their attack is more common on eggplant and okra (Singh and Mukherjee 1991), the ensuing plant injuries causing a reduction in crop yield. Dhooria and Bindra (1980) recorded the percentage reduction of the *T. urticae* population after two, seven, 14, and 22 days of treatment on eggplant and found that spray containing 0.025% oxydemeton—methyl proved most effective, followed by 0.03% phosphamidon, 0.06% chlorobenzilate, 0.025% diazinon, 0.025% binapacryl, and 0.025% dicofol. They recommended oxydemeton—methyl for the control of *T. urticae* because of its high residual activity, quick knock—down effect, and availability. However, in another study dicofol 0.05% was found to be the most effective causing 70.56 to 91.85% reduction of mites in okra and 66.99 to 99.20% reduction in eggplant (Ramaraju 2004). The insecticides such as wettable sulphur, phosalone, and monocrotophos were next in order in controlling spider mite spread. The botanicals such as NOPO (Neem oil + Pungam oil), NSKE (Neem seed kernel extract), and TNAU NO (Tamil Nadu Agricultural University Neem oil) were also found effective in descending order by the same group.
